# Supplementary material for: Human rhinovirus promotes STING trafficking to replication organelles to promote viral replication
Source: Nat Commun. 2022 Mar 17;13:1406. doi: 10.1038/s41467-022-28745-3 (PMC8931115; doi:10.1038/s41467-022-28745-3)
Supplement: Supplementary file 1 — Supplemental Information [file 41467_2022_28745_MOESM1_ESM.pdf]

Supplemental information

Human rhinovirus promotes STING trafficking to replication organelles to promote viral replication

Martha Triantafilou[1,2] *, Joshi Ramanjulu[3], Lee Booty[1], Gisela Jimenez-Duran[1,2], Hakan Keles[4], Ken Saunders[5], Neysa Nevins[,3], Emma Koppe[1], Louise K. Modis[5], Scott Pesiridis[3], John Bertin[3] and Kathy Triantafilou[1, 2]

*Author for correspondence: Dr Martha Triantafilou, email: TriantafilouM@cardiff.ac.uk

Supplementary Figure 1. STING concentrates in PI4P rich replication organelles.

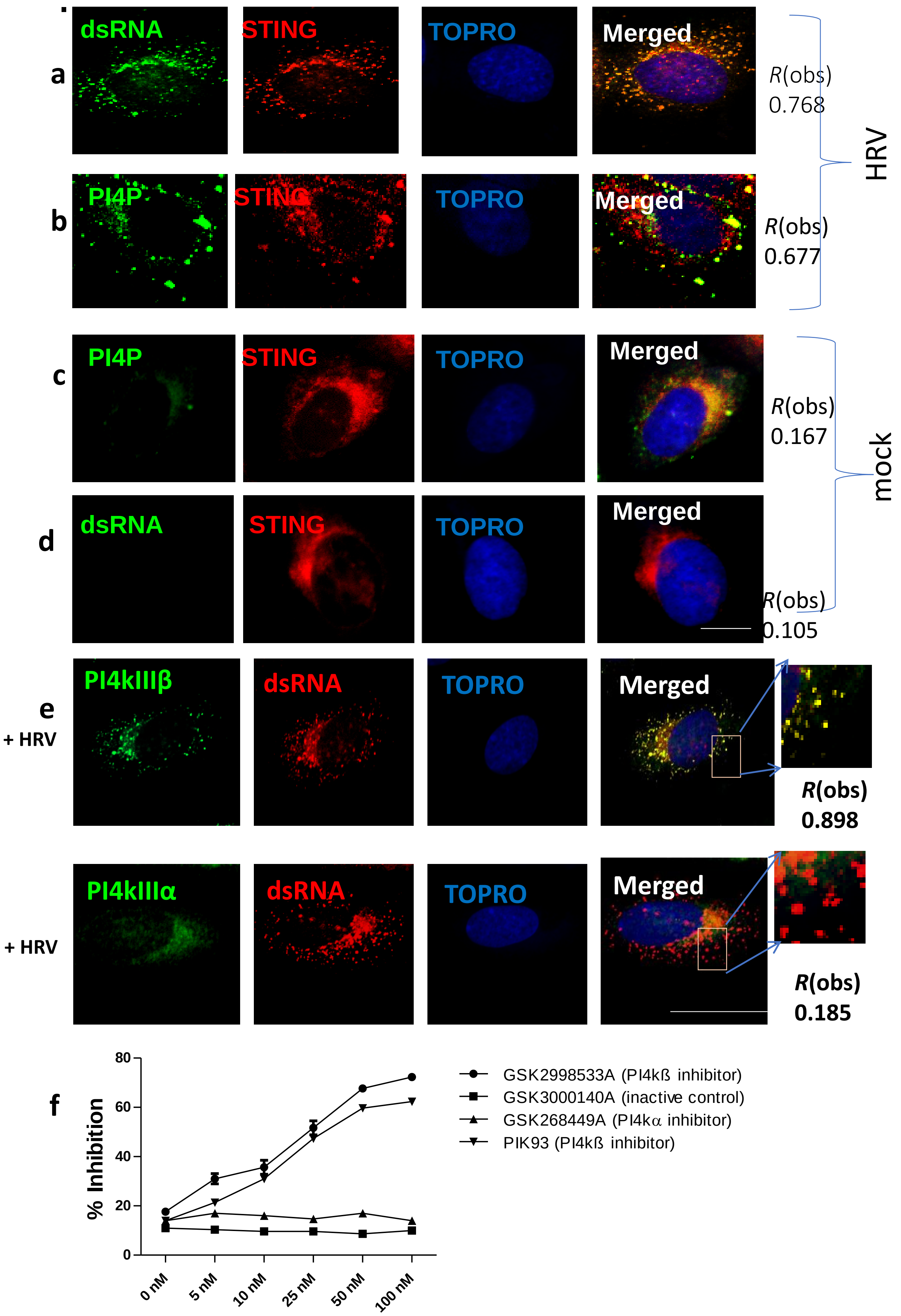

BEAS-2B cells infected with HRV-A1B (MOI 10) for 4 h (**a**,**b**). Uninfected cells were also used as a control (**c**). Cells were labelled for PI4P, a mAb specific for dsRNA (J2) and STING (**d**). The nucleus was stained with TOPRO-3. Bars shown are 10 µm. The degree of colocalisation *R*(obs) was determined using ImageJ software via the Costes' method. BEAS-2B cells infected with HRV-A1B (MOI 10) for 4 h were assessed for PI4KIIIα and PI4KIIIβ colocalisation with dsRNA (**e**). As in (**e**), BEAS-2B cells were infected with HRV-A1B in the presence of GSK2998533 (100 nM), GSK2683449 (100 nM) or PIK93 (1 uM) and HRV replication assessed in a dose-response titration by qPCR (**f**) Data is representative of at least 20 technical replicates taken from 3 biological repeats.

Supplementary Figure 2. HRV infection causes depletion of ER Ca^{2+} stores and translocation of STIM1 into junctional areas between the ER and plasma membrane.

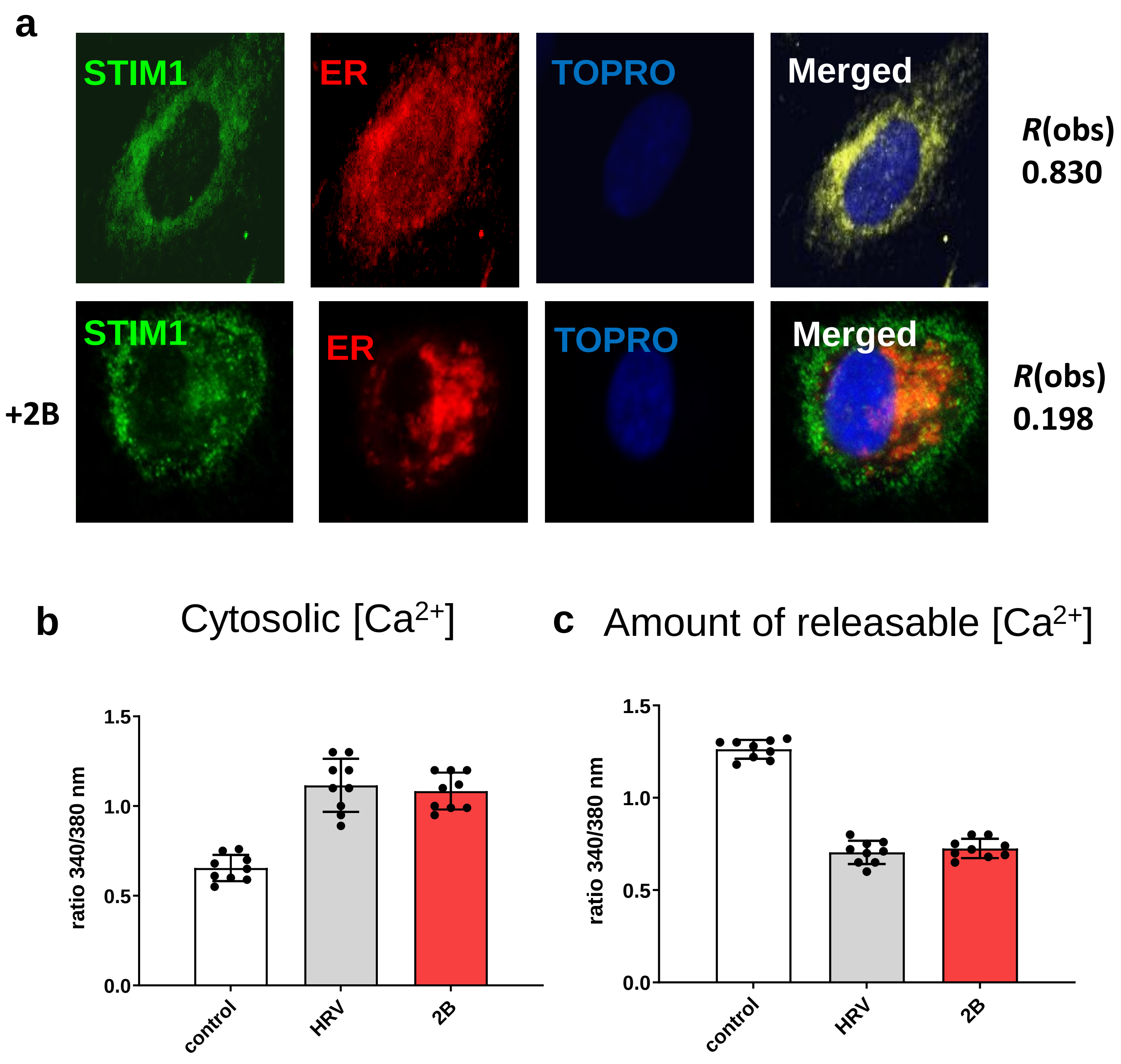

Cells were stained for STIM1 (green), ER/calreticulin (red) and nuclei (TOPRO) under resting conditions (mock) or when infected with HRV (**a**). The images showed that STIM1 is located in the ER but upon expression of 2B –myc, it traffics between the ER and the plasma membrane. Bars shown are 10 mm. The degree of colocalisation *R*(obs) was determined using ImageJ software via the Costes' method. Data is representative of at least 20 technical replicates taken from 3 biological repeats.Fura2-based calcium measurements of cytosolic calcium (**b**) and releasable calcium (**c**) upon infection with HRV or transfection of p2B-myc. Fluorescence was measured every 2 s with the excitation wavelength alternating between 340 and 380 nm and the emission fluorescence being recorded at 492 nm and the ratio of 340/380 was calculated. Expression of 2B resulted in a significant increase in cytosolic Ca^{2+} . Data are means ± SD (n=3, from three independent experiments) (**b**). To determine whether rhinovirus 2B protein affects the intracellular stores of ER and Golgi, BEAS-2B cells were treated with thapsigargin (TG) (1 µM), an inhibitor of endoplasmic reticulum Ca^{2+} ATPase pumps leading to an accumulation of Ca^{2+} in the cytosol, and Fura2 was used to measure the cytosolic calcium level as described above. Our results showed that cells expressing 2B-myc exhibited a significant decrease in the amount of TG-releasable Ca^{2+} . Data is mean+/-SD (n=3, independent experiments). (**c**).

Supplementary Figure 3. STING interacts with PI4P.

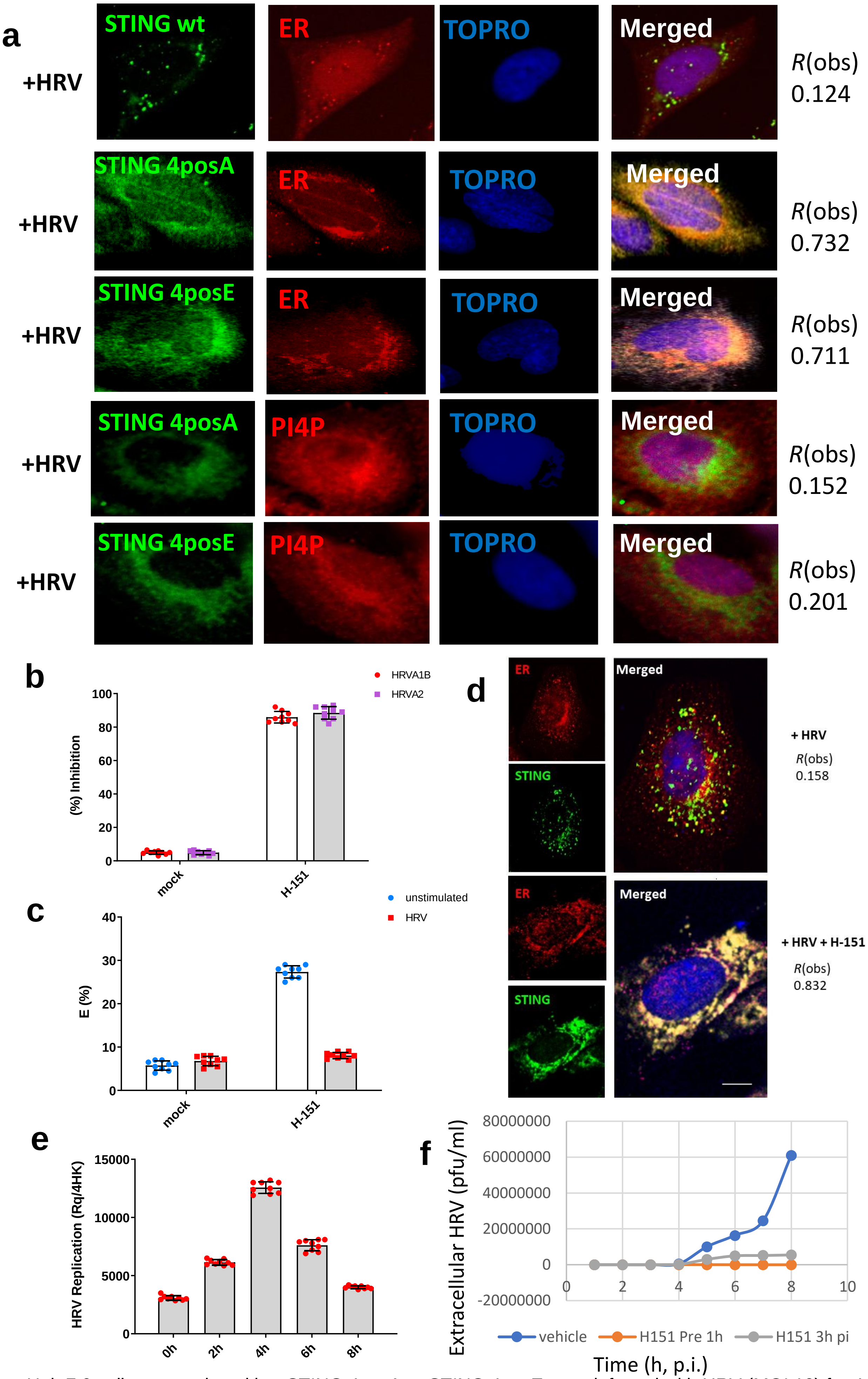

Huh 7.0 cells expressing either STING 4posA or STING 4posE were infected with HRV (MOI 10) for 4 h and stained for STING 4posA or STING 4posE (green) and calreticulin as an ER marker (red) or PI4P (red). Bars shown are 10 µm. The degree of colocalization *R*(obs) was determined using ImageJ software via the Costes' method. Data is representative of three independent experiments with at least 20 technical replicates. (**a**). BEAS-2B cells were infected with either HRV-A1B, or HRV-A2 and/or were treated with STING antagonist H-151 (1µM) and percentage inhibition is shown. Data are means ± SD, n = 3, independent experiments (**b**).FRET efficiency (E%) is also shown between STING and PI4P in the absence and presence of STING antagonist H-151 (1µM). Data is mean+/-SD (n=3, independent experiments). (**c**).Confocal microscopy images of ER and STING in BEAS-2B cells infected with HRV-A1B (4 hr p.i.) in the absence or presence of 10nM STING antagonist H-151 are shown. The images display that STING moves away from the ER (there is no colocalization) upon infection. When cells are treated with the STING antagonist STING resides in the ER (there is significant colocalization). Data is representative of three independent experiments with at least 20 technical replicates.(**d**). Quantitative PCR measurement of the viral genome during replication in the supernatants of cells is shown (**e**). Infectious virus at various times post infection (p.i.) in plaque forming units (pfu/ml) in the presence and absence of H151. Data are means ± SD, n = 3, independent experiments (**f**).

Supplementary Figure 4. At the later stages of HRV infectious cycle, STING is localised in large autophagosome-like vesicles which are distinct from replication organelles.

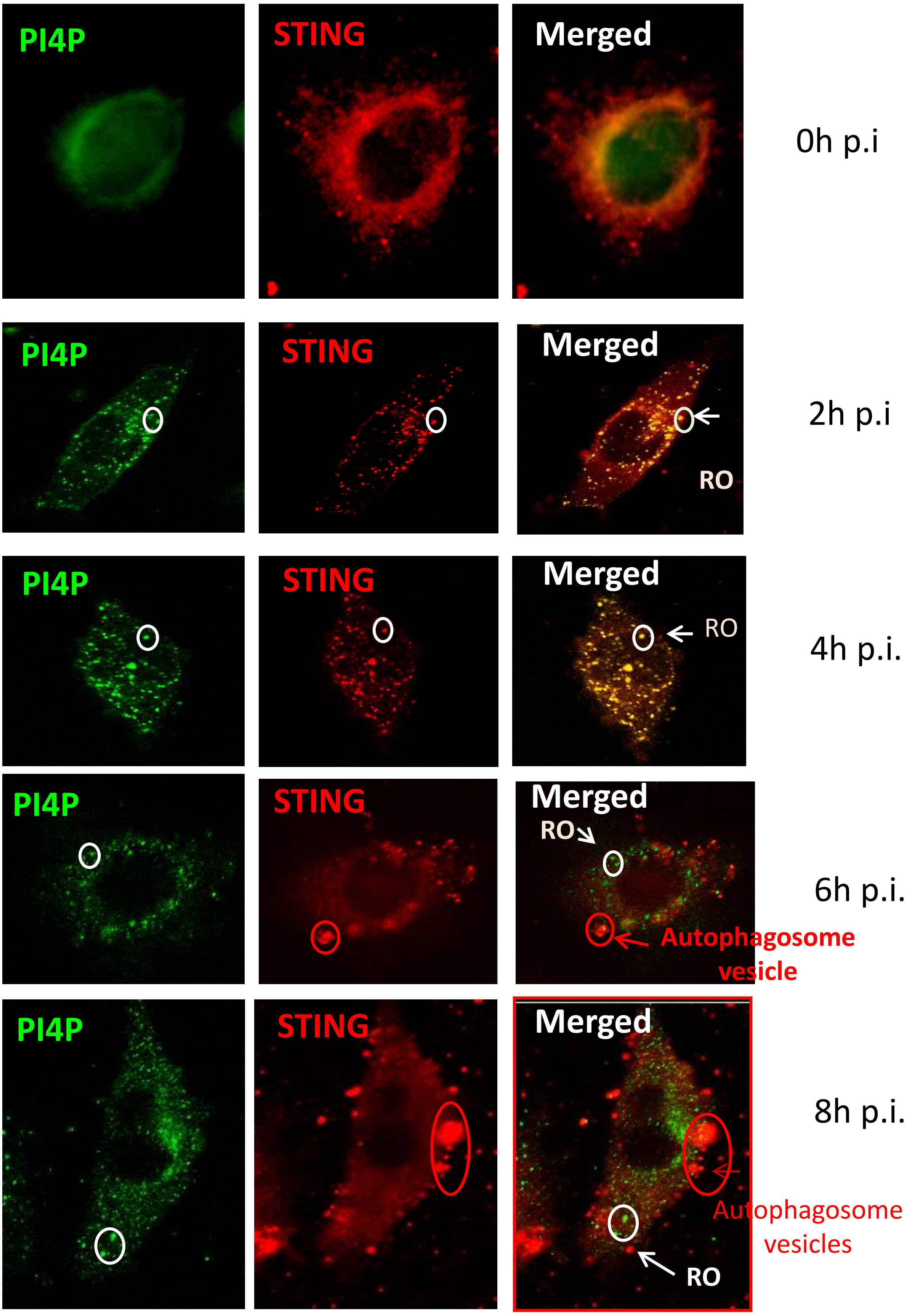

Bronchial epithelial cells were infected with HRV-A1B and a time course of STING and PI4P trafficking was performed using confocal microscopy. The micrographs show that between 2-4hp.i STING colocalises with PI4P in RO. Then between 6-8hr p.i. 80% of STING trafficked to large vesicles distinct from PI4P rich RO. Data is representative of three independent experiments with at least 20 technical replicates.

Supplementary Figure 5. Increase in the overall lipid signal during HRV infection.

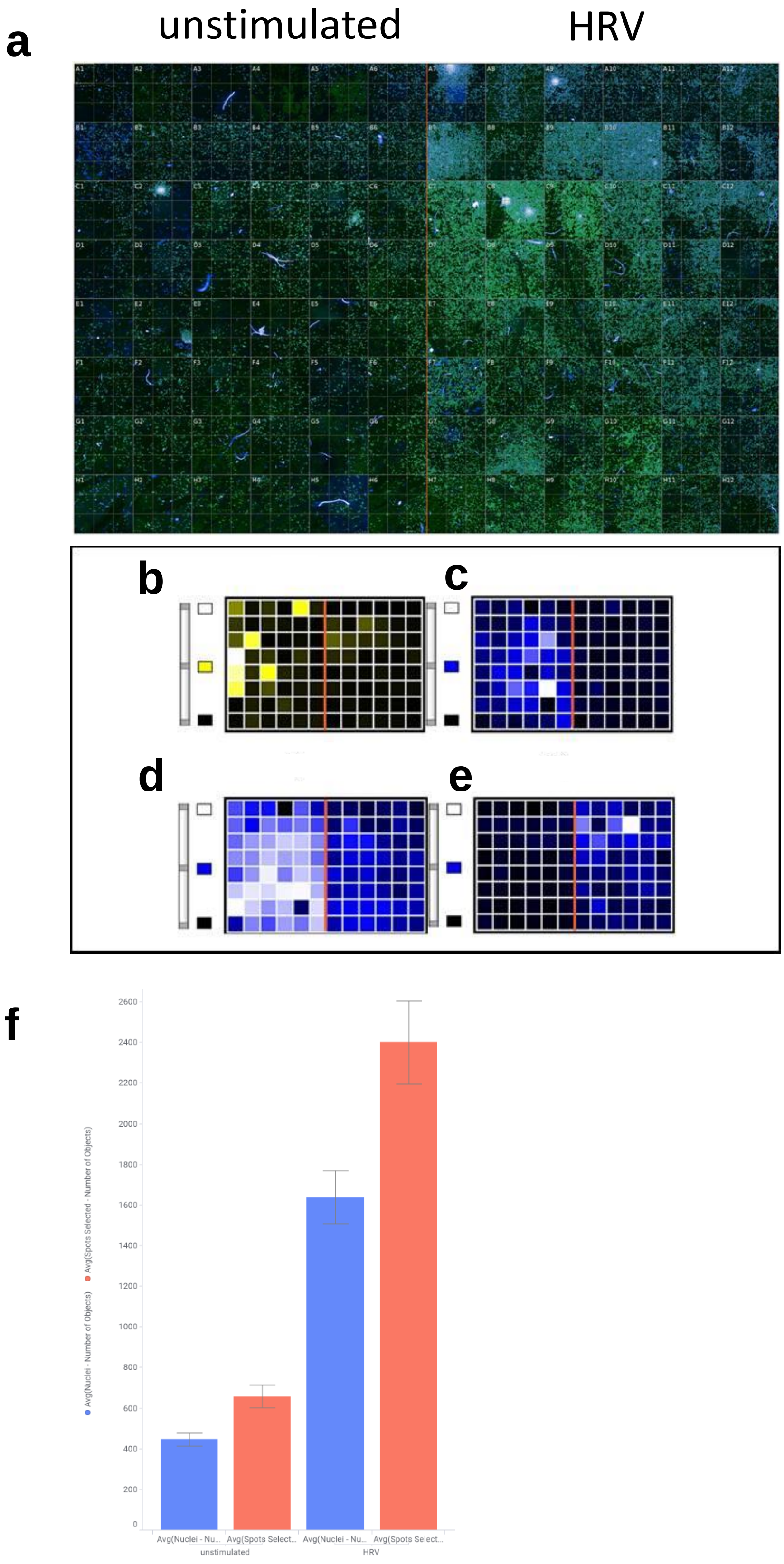

PS lipid (green)(labelled with an anti-PS Alexa488 antibody) is re-distributed leading to an increase in the overall lipid signal. In addition, the number of nuclei is noticeably increased post infection. This is expected since nuclei are more prominent in autophagy and the virus in our case forms autophagosome like vesicles. The nuclei were labelled with Hoechst (blue), in the unstimulated cells on the left half and infected cells on the right half of the plate, respectively. Each location label in each of the 3x3 mosaics represents an individual well in the plate (**a**). False colour heat map of the 96 well plate where white indicates highest and black indicates lowest value of (**b**) average lipid area size, (**c**) average width to length ratio of the lipid segments, (**d**) roundness of segmented lipid regions and (**e**) detected number of nuclei per well as shown in each square. Control cells and infected cells are represented on the left and right half of the plate, respectively, as separated by the orange bar in the middle of each map. Bar charts of the average number of nuclei and spots per well (**f**) (means±SD) (n=3, from three independent experiments)), per condition.

Supplementary Figure 6. Confocal/DIC images Alexa 488-annexin-V PS vesicle release from plasma membrane at 7hr p.i.

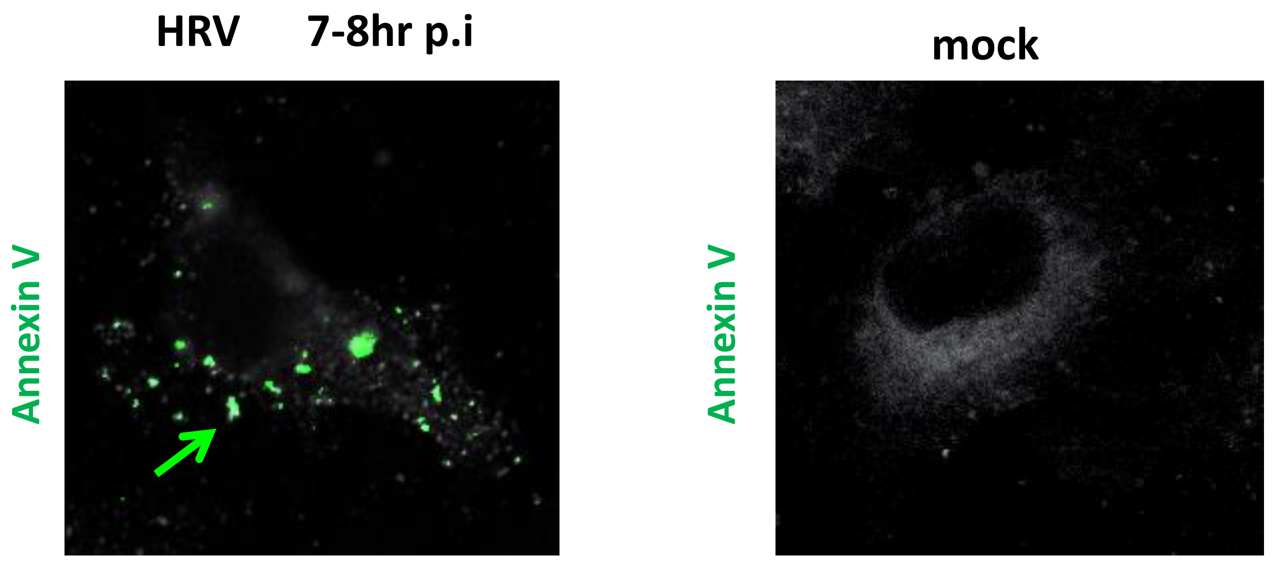

Confocal/DIC images Alexa 488-annexin-V PS vesicle release from plasma membrane at 7hr p.i. Data is representative of three independent experiments with at least 20 technical replicates.

Supplementary Figure 7. STING-STIM interactions

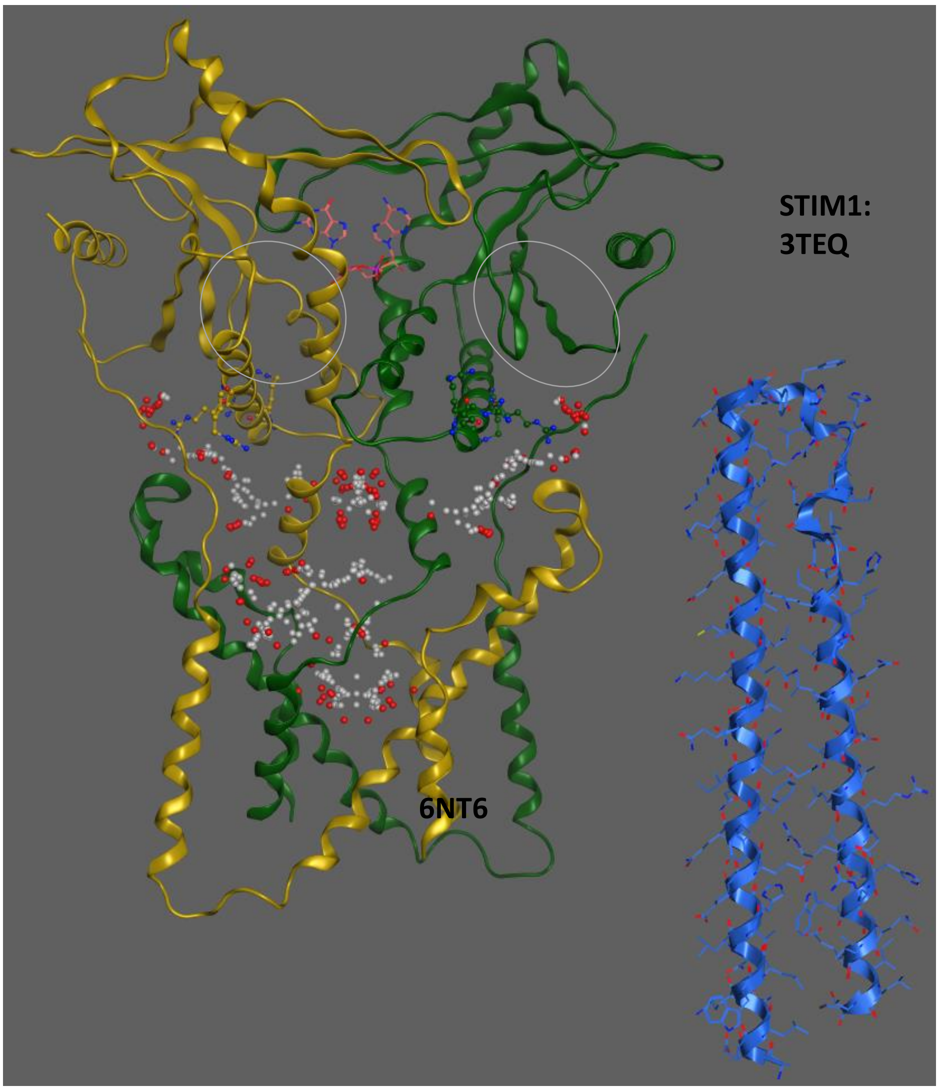

Comparison of chicken STING (6NT7) and fragment of human STIM1 (blue, residues 344-438 shown). Positively charged residues (circled): Chicken: R286, R289, R294, R298; Human: R281, R284, K289, R293; STIM1 (blue, residues 344-438 shown) can inhibit STING.

Supplementary Figure 8. Proposed PI4P interaction site.

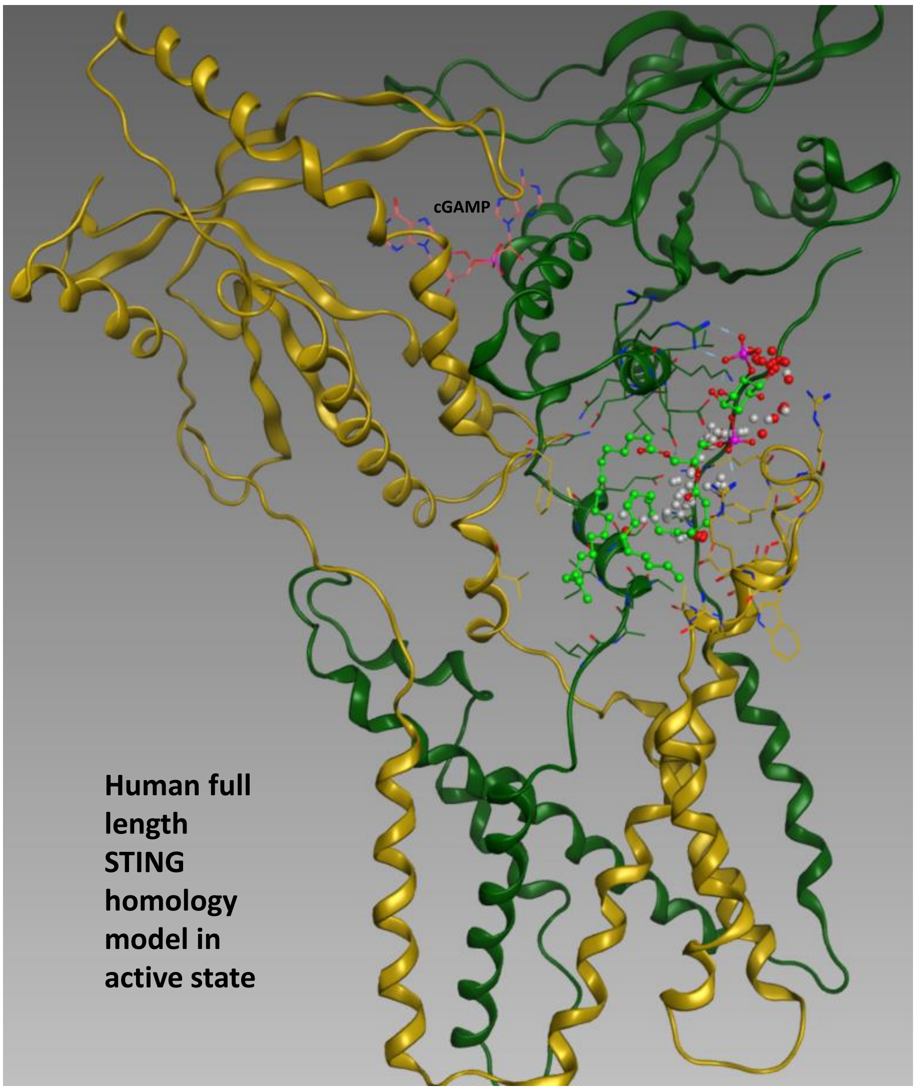

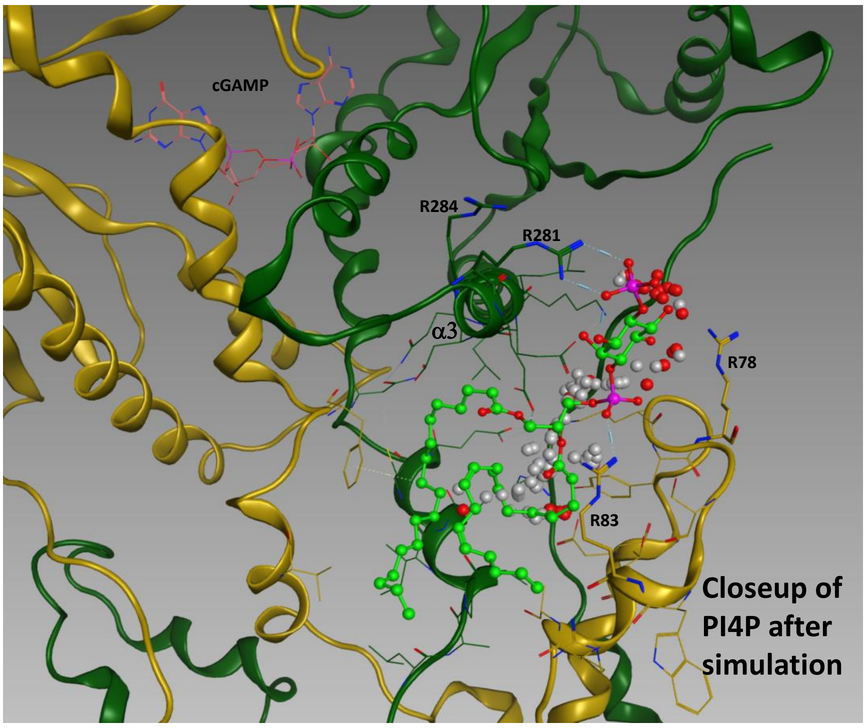

A human STING homology model was built using the N-terminal domain of chicken STING (6NT7) and CTD of human STING (6DXG). PI4P was modeled near R281/284 in one of the SiteFinder identified pockets from full length chicken STING (6NT7). Molecular dynamics was carried out for 100 ns in a 10 A box of water using default conditions with Desmond in Maestro 2020.1.

Table 1. Primers used in the study

**HRVP3 Fw**	**Applied Biosystems**	**GTTGTTCCCACCCAGATCAC**
**HRVP3 Rv**	Applied Biosystems	CTCAGTTGTTTTTGCCCTTGTGG
**HRVP6 Fw**	Applied Biosystems	TGGACAAGGTGTGAAGAGCC
**HRVP6 Rv**	Applied Biosystems	ACACGGACACCCAAAGTAGT
**HRV-C15 Fw**	Applied Biosystems	CCTCCGGCCCCTGAATG
**HRV-C15 Rv**	Applied Biosystems	AAACACGGACACCCAAAGTAGT
**TBP primers**	Applied Biosystems	Hs00427620_m1
**UBB primers**	Applied Biosystems	Hs00430290_m1
**B2M primers**	Applied Biosystems	Hs00187842m1

Table 2. gRNA sequences used with CRISPR/Cas9 Knockout (KO) Plasmid

Guide 1	Guide 2
MDA5 gRNA Target sequence CGAATTCCCGAGTCCAACCA	gRNA Target sequence AGCGTTCTCAAACGATGGAG
STING gRNA Target sequence GCGGGCCGACCGCATTTGGG	gRNA Target sequence GGTGCCTGATAACCTGAGTA
RIG-I gRNA Target sequence GGGTCTTCCGGATATAATCC	gRNA Target sequence TTGCAGGCTGCGTCGCTGCT
IRF3 gRNA Target sequence ATCAGGCCCATGCCCGCTGT	gRNA Target sequence GGGAGTGGGATTGTCCAAGC
TBK1 gRNA Target sequence CATAAGCTTCCTTCGTCCAG	gRNA Target sequence ATCACTTCTTTATTCCTACG
