# Supplementary material for: Human rhinovirus promotes STING trafficking to replication organelles to promote viral replication
Source: Nat Commun. 2022 Mar 17;13:1406. doi: 10.1038/s41467-022-28745-3 (PMC8931115; doi:10.1038/s41467-022-28745-3)
Supplement: Supplementary file 3 — Reporting Summary [file 41467_2022_28745_MOESM3_ESM.pdf]

nature research

Corresponding author(s): Martha Triantafilou

Last updated by author(s): 2021/02/10

Reporting Summary

Nature Research wishes to improve the reproducibility of the work that we publish. This form provides structure for consistency and transparency in reporting. For further information on Nature Research policies, see our Editorial Policies and the Editorial Policy Checklist.

Statistics

For all statistical analyses, confirm that the following items are present in the figure legend, table legend, main text, or Methods section.

n/a	Confirmed	
☐	☒	The exact sample size (*n*) for each experimental group/condition, given as a discrete number and unit of measurement
☐	☒	A statement on whether measurements were taken from distinct samples or whether the same sample was measured repeatedly
☐	☒	The statistical test(s) used AND whether they are one- or two-sided<br>*Only common tests should be described solely by name; describe more complex techniques in the Methods section.*
☒	☐	A description of all covariates tested
☒	☐	A description of any assumptions or corrections, such as tests of normality and adjustment for multiple comparisons
☒	☐	A full description of the statistical parameters including central tendency (e.g. means) or other basic estimates (e.g. regression coefficient) AND variation (e.g. standard deviation) or associated estimates of uncertainty (e.g. confidence intervals)
☐	☒	For null hypothesis testing, the test statistic (e.g. *F*, *t*, *r*) with confidence intervals, effect sizes, degrees of freedom and *P* value noted<br>*Give P values as exact values whenever suitable.*
☒	☐	For Bayesian analysis, information on the choice of priors and Markov chain Monte Carlo settings
☒	☐	For hierarchical and complex designs, identification of the appropriate level for tests and full reporting of outcomes
☐	☒	Estimates of effect sizes (e.g. Cohen's *d*, Pearson's *r*), indicating how they were calculated

Our web collection on statistics for biologists contains articles on many of the points above.

Software and code

Policy information about availability of computer code

Data collection	ZenBlack
Data analysis	ZenBlack, Image J Java 1.8.0_172, Graphpad Prism 9.0

For manuscripts utilizing custom algorithms or software that are central to the research but not yet described in published literature, software must be made available to editors and reviewers. We strongly encourage code deposition in a community repository (e.g. GitHub). See the Nature Research guidelines for submitting code & software for further information.

Data

Policy information about availability of data

All manuscripts must include a data availability statement. This statement should provide the following information, where applicable:

- Accession codes, unique identifiers, or web links for publicly available datasets
- A list of figures that have associated raw data
- A description of any restrictions on data availability

The datasets generated during and/or analysed during the current study are available from the corresponding author on reasonable request. Source data are provided with this manuscript. GTEx database phs000424.v8.p2 was used in this study.

Field-specific reporting

Life sciences study design

All studies must disclose on these points even when the disclosure is negative.

Sample size	Sample size was determined by a statistician. Three biological and three technical replicates were performed.
Data exclusions	No data were excluded from the analysis
Replication	Each experiment was performed at least three times. All data were reproducible.
Randomization	Wells with confluent cells were randomly assigned to one of the N treatment conditions for every experiment
Blinding	Experimenters were blinded to the treatment conditions during the analysis. Partial blinding was performed: No blinding during the conduct of an experiment but proper blinding during analysis

Reporting for specific materials, systems and methods

We require information from authors about some types of materials, experimental systems and methods used in many studies. Here, indicate whether each material, system or method listed is relevant to your study. If you are not sure if a list item applies to your research, read the appropriate section before selecting a response.

Materials & experimental systems

n/a	Involved in the study	
☐	☒	Antibodies
☐	☒	Eukaryotic cell lines
☒	☐	Palaeontology and archaeology
☒	☐	Animals and other organisms
☒	☐	Human research participants
☒	☐	Clinical data
☒	☐	Dual use research of concern

Methods

n/a	Involved in the study	
☒	☐	ChIP-seq
☒	☐	Flow cytometry
☒	☐	MRI-based neuroimaging

Antibodies

Antibodies used	All antibodies used and suppliers are written in Materials and Methods section of the manuscript. Anti-STING rabbit polyclonal antibody (Invitrogen, PA5-23381, 1:100 IF)), was purchased from Invitrogen. Aanti-STING goat antibody (SantaCruz, sc241046, 1:100 iF)) was purchased from Santa cruz., J2 mAb was obtained from (English & Scientific Consulting, 1:100 IF). PI4P IgM mAb was obtained from (Echelon, ZP004-2, 1:100 IF)), Anti Calreticulin goat polyclonal (ThermoFisher, PA1-33045, 1:100 IF) was obtained from (Thermofisher Scientific and) was used to stain Endoplasmic reticulum. ERGIC-53 monoclonal antibody (ENZO, OTI1A8, 1:100 IF) from ENZO as well as ERGIC-53 monoclonal antibody (Sigma-Aldrich, E1031, 1:100 IF) from Sigma-Aldrich were used. Alexa Fluor-488 or -546 labeledlabelled secondary antibodies against mouse or rabbit IgG or IgM (Thermofisher Scientific, 1:100 IF) were used.
Validation	All antibodies used were validated by the supplier.

Eukaryotic cell lines

Policy information about cell lines

Cell line source(s)	Bronchial epithelial cells (BEAS-2B, ATCC cat. Number CRL-9609). ALIs (Air Liquid interface) of airway epithelial cells were grown on transwells from normal donors (Epithelix, NHBE – Human Bronchial Epithelial Cells, Donor 28060 p3, and Donor 25265COPD Donor 63 p3)
Authentication	None of the cell lines used were authenticated
Mycoplasma contamination	The cell lines were tested and were shown to be negative for mycoplasma contamination
Commonly misidentified lines (See ICLAC register)	No commonly misidentified cell lines were used in the study
